# Promoting smoking cessation in Pakistani and Bangladeshi men in the UK: pilot cluster randomised controlled trial of trained community outreach workers

**DOI:** 10.1186/1745-6215-12-197

**Published:** 2011-08-19

**Authors:** Rachna A Begh, Paul Aveyard, Penney Upton, Raj S Bhopal, Martin White, Amanda Amos, Robin J Prescott, Raman Bedi, Pelham Barton, Monica Fletcher, Paramjit Gill, Qaim Zaidi, Aziz Sheikh

**Affiliations:** 1 UK Centre for Tobacco Control Studies, Primary Care Clinical Sciences, University of Birmingham, Birmingham, B15 2TT, UK; 2 Psychological Sciences, Institute of Health and Society, University of Worcester, Worcester, WR2 6AJ, UK; 3 Ethnicity and Health Research Group, Centre for Population Health Sciences, University of Edinburgh, Edinburgh, EH8 9AG, UK; 4 Fuse, UKCRC Centre for Translational Research in Public Health, Institute of Health & Society, Newcastle University, NE2 4HH, UK; 5 UK Centre for Tobacco Control Studies, Centre for Population Health Sciences, University of Edinburgh, Edinburgh, EH8 9AG, UK; 6 International Centre for Child Oral Health, King's College London, London, WC2B 5RL, UK; 7 Health Economics, University of Birmingham, Birmingham, B15 2TT, UK; 8 Education for Health, Warwick, CV34 4AB, UK; 9 British Heart Foundation, London, W1H 6DH, UK; 10 Allergy & Respiratory Research Group, Centre for Population Health Sciences, University of Edinburgh, Edinburgh, EH8 9AG, UK; 11 CAPHRI, University of Maastricht, The Netherlands

## Abstract

**Background:**

Smoking prevalence is high among Pakistani and Bangladeshi men in the UK, but there are few tailored smoking cessation programmes for Pakistani and Bangladeshi communities. The aim of this study was to pilot a cluster randomised controlled trial comparing the effectiveness of Pakistani and Bangladeshi smoking cessation outreach workers with standard care to improve access to and the success of English smoking cessation services.

**Methods:**

A pilot cluster randomised controlled trial was conducted in Birmingham, UK. Geographical lower layer super output areas were used to identify natural communities where more than 10% of the population were of Pakistani and Bangladeshi origin. 16 agglomerations of super output areas were randomised to normal care controls vs. outreach intervention. The number of people setting quit dates using NHS services, validated abstinence from smoking at four weeks, and stated abstinence at three and six months were assessed. The impact of the intervention on choice and adherence to treatments, attendance at clinic appointments and patient satisfaction were also assessed.

**Results:**

We were able to randomise geographical areas and deliver the outreach worker-based services. More Pakistani and Bangladeshi men made quit attempts with NHS services in intervention areas compared with control areas, rate ratio (RR) 1.32 (95%CI: 1.03-1.69). There was a small increase in the number of 4-week abstinent smokers in intervention areas (RR 1.30, 95%CI: 0.82-2.06). The proportion of service users attending weekly appointments was lower in intervention areas than control areas. No difference was found between intervention and control areas in choice and adherence to treatments or patient satisfaction with the service. The total cost of the intervention was £124,000; an estimated cost per quality-adjusted life year (QALY) gained of £8,500.

**Conclusions:**

The intervention proved feasible and acceptable. Outreach workers expanded reach of smoking cessation services in diverse locations of relevance to Pakistani and Bangladeshi communities. The outreach worker model has the potential to increase community cessation rates and could prove cost-effective, but needs evaluating definitively in a larger, appropriately powered, randomised controlled trial. These future trials of outreach interventions need to be of sufficient duration to allow embedding of new models of service delivery.

**Trial registration:**

Current Controlled Trials ISRCTN82127540

## Background

There are marked ethnic and gender variations in smoking prevalence in the UK. In 2004, smoking prevalence was 40% in Bangladeshi men and 29% in Pakistani men compared with 24% in the general male population [1]. Stopping smoking is especially important in Pakistani and Bangladeshi groups because the incidence of heart disease, stroke and type 2 diabetes is higher than in other population groups [2,3], and stopping smoking would reduce the risk of these diseases by more than a third [4,5]. The UK has a national network of National Health Service (NHS) smoking cessation services offering interventions proven to be effective in facilitating smoking cessation [6-10]. Overall, only about 3% of smokers use the NHS stop smoking services (SSS), although over 40% try to quit without use of the SSS each year [11]. At the time of planning the trial, South Asian groups (i.e. mainly those of Indian, Pakistani, and Bangladeshi origin) were half as likely to use the cessation services than the rest of the population [12]. There might be specific cultural beliefs that deter Bangladeshi and Pakistani smokers from using NHS cessation services. Qualitative research has shown that Bangladeshi and Pakistani adults were well aware of the dangers of smoking and were motivated to quit, but tended to focus on using willpower and were uncertain about the value of support and cessation medications [13]. There is no recognised and implemented model that has been shown to challenge these types of beliefs about using the NHS cessation services.

A systematic review reported that interventions to increase service uptake and rates of cessation among disadvantaged groups have shown varying success [14]. One study found that proactively identifying smokers through primary care practice records and providing these smokers with brief advice and referral to a smoking cessation advisor increased contacts with the services and the number of quit attempts, but did not increase cessation rates [15]. Two studies included in the review [14] focused primarily on interventions designed to increase service uptake and cessation in minority ethnic groups [16,17]. One uncontrolled before-after study used social marketing, tailored to cultural beliefs, to highlight the dangers of smoking in Turkish and Kurdish communities in London [16]. The follow-up survey showed that out of 142 respondents (47% of original sample), half had recognised the advertising materials used in the campaign and 13% reported that they had given up smoking. The second study randomised African American communities in the US to a marketing campaign of adverts, posters and outreach aimed at increasing calls to a Cancer Information Service quit line or to control [17]. The volume of calls from African Americans to the quit line was significantly higher in experimental communities (558 calls) than in control communities (7 calls, P < 0.008), but no data on quitting were presented.

We undertook a pilot trial of an intervention designed to offer a culturally tailored, trained community smoking cessation worker model of care. A Cochrane review reported that community lay health workers have been effective in primary care, promoting the uptake of immunisation and for improving outcomes for selected infectious diseases in comparison with usual care [18]. Only one randomised controlled trial included in the review examined the use of lay workers in encouraging smokers to quit [19]. In this US study, 22 church communities were randomly allocated to either an intensive intervention involving the use of smoking cessation lay workers or distribution of self-help materials only. The study reported no difference in quit rates, but the intervention communities were more likely to intend to quit in the future. Similarly, another recent review [20] that examined the impact of lifestyle advisors on health improvement identified four randomised controlled trials where lay advisors were used to promote smoking cessation [21-24]. Three of these studies found that lay advisor interventions improved quit rates compared to the control [22-24]. One study involved lay health workers delivering home-based smoking cessation programmes, tailored specifically to the cultural beliefs and practices of Latino smokers in the US [24]. One week abstinence rates were twice as high in the intervention group (20.5%) compared to a helpline control group (8.7%, P < 0.005). In the UK, NHS SSS have developed community outreach interventions with lay workers, but these are currently not evaluated. These include facilitators aiming to improve access to existing mainstream smoking cessation services and the development of parallel home-based specialist services.

Building on these wider insights, we developed and piloted a model of community SSS for Bangladeshi and Pakistani male smokers and their wider communities. We focused on men because the prevalence of smoking in these ethnic groups is substantially higher than in women [1]. Also, the stigma of Pakistani and Bangladeshi women smoking [25] means that these women rarely present for treatment [12].

We describe here the quantitative outcomes and processes involved in this pilot cluster randomised controlled trial of trained community outreach workers. The aim was to examine whether the intervention led more Pakistani and Bangladeshi men to stop smoking with NHS support compared to standard care. We also assessed whether the intervention had an impact on the type of treatments chosen, adherence to treatments, attendance at clinic appointments and patient satisfaction with the service. We in addition conducted a longitudinal qualitative study in parallel with the trial to explore the approach outreach workers took when recruiting service users and supporting smoking cessation, and to explore how their role and the intervention changed over time; the results from this qualitative work are reported in detail elsewhere [26,27].

## Methods

### Study design

This was an exploratory Phase II cluster randomised controlled trial, as defined by the MRC Framework for Complex Interventions [28-30]. The setting was Birmingham East and North Primary Care Trust (BEN PCT) and the Heart of Birmingham Teaching Primary Care Trust (HoB tPCT). A detailed trial protocol has been published [31]. It was designed to test the acceptability and feasibility of the intervention and the development of the intervention during the trial was part of the approach [26]. It was also designed to assess the feasibility and acceptability of the trial methods. It was not designed to provide definitive evidence of efficacy and hence we therefore did not undertake any sample size calculations.

We randomised natural communities to either standard behavioural support and medication available in NHS clinics (internal control), or to the same service augmented by community-based outreach workers, aiming to encourage and support male Pakistani and Bangladeshi smokers to quit smoking (intervention). We selected these areas to be as widely dispersed as possible, but they were still geographically close and hence it was possible that this would lead to contamination (i.e. the beneficial effects of outreach also being seen in control communities). Consequently, we measured the outcome variables in all other Pakistani and Bangladeshi men in other areas of HoB and BEN PCTs (external control); some of these areas were a reasonable distance from the intervention and internal control areas and were judged unlikely to experience contamination.

We used two different approaches to collect our outcome and process data. We obtained anonymised data on all Pakistani and Bangladeshi residents, aged 18 years or over in our intervention, control and external control areas that used an NHS SSS. These data, collected routinely by the NHS SSS, contained information on the service users' age, ethnicity, postcode, quit date and smoking status at four weeks after the quit date. The NHS SSS tried to contact all service users who were abstinent at four-weeks in our intervention, control and external control areas for verification of quit status at three-month and six-month follow-up.

We collected more detailed process data on service use patterns and on satisfaction with the service from a sample of clinics operating in the intervention, control and external control areas. These clinics were chosen because they had treated several Pakistani and Bangladeshi smokers prior to the study. All participating service providers were given a pack containing a brief procedure guide, information sheets, consent forms, data collection forms and a method to contact the research team. Pakistani and Bangladeshi smokers aged 18 years or over were asked to participate by their service provider during routine consultation. These data were not anonymised and hence service users gave their consent to give the data. Service providers recorded weekly attendance, choice of treatments and adherence (where adherence was defined as good or less than good for each type of treatment). At three-month follow-up, service users were contacted by NHS SSS to collect information on their experiences of using the service, which was recorded on a patient satisfaction questionnaire developed by the research team.

### Randomisation

Census lower layer super output areas (LSOAs) were used as the unit of allocation [32]. LSOAs are the smallest unit of census geography consisting of 400 households on average. LSOAs within the two PCTs, where the combined Pakistani and Bangladeshi population was more than 10% of the total, were mapped. Contiguous LSOAs were aggregated into natural communities (i.e. areas where people live, work, shop, etc.) using local knowledge. We created buffer zones around the trial areas to reduce the risk of contamination.

There were eight agglomerations of LSOAs in which more than 30% of the population were Pakistani and Bangladeshi and eight low density areas where 10-29% of the population from these groups lived. The 16 areas were stratified, firstly by the proportion of Pakistani and Bangladeshi residents and secondly, by absolute population size into two further strata. The trial statistician used permuted blocks of four to randomise eight areas to intervention and eight to control. Despite the stratification for size, the total resident population of the control areas was much larger than in the intervention areas. The managers of the NHS SSS were unhappy to work on the smaller target population; therefore, with the agreement of the Independent Trial Steering Committee, we swapped the intervention and control areas status prior to the intervention starting. Maps of the final areas and their allocation to the two trial arms are published in our trial protocol [31].

As socio-economic position is a strong predictor of smoking status, mean Index of Multiple Deprivation (IMD) scores were calculated for each area to rule out potential confounders. The IMD is a LSOA based measure of deprivation across England. Scores are calculated using multiple indices of deprivation within seven domains: income, employment, health and disability, education skills and training, barriers to housing and services, living environment, and crime [33].

### Intervention group

Four male, community based, stop smoking advisors (SSAs), known henceforth as 'outreach workers', provided additional support to NHS SSS, which was otherwise similar to that provided in the control areas. Two outreach workers were of Bangladeshi origin and two of Pakistani origin. Between them, they spoke the main relevant languages (i.e. Sylheti, Bengali, Punjabi, Mirpuri, Urdu, and English). The outreach workers were paired into two teams of one Bangladeshi and one Pakistani outreach worker in each PCT. Two outreach workers had worked as SSAs prior to the study. One Pakistani outreach worker resigned after six months and was replaced by another Pakistani SSA.

Outreach workers had two weeks of training in delivering behavioural support and medication management for smoking cessation, general health promotion, communication skills, and the cultural specific norms of Pakistani and Bangladeshi smokers. The training involved role-playing the activities in outreach in English and in minority languages. All outreach workers were assessed as competent based on these role-plays by the end of training. The training was delivered by accredited NHS trainers and the research team.

Two local stop smoking service managers from HoB tPCT and BEN PCT (referred to henceforth as the 'management team') supervised the outreach workers. The outreach workers and managers met fortnightly initially then monthly during which the diaries and experience of the outreach workers were reviewed and plans made.

The intervention was delivered in two phases, although this was unplanned at the outset. During the first phase, i.e. November 2007 to May 2008, outreach workers concentrated on referring people to existing services that included pharmacies, drop-in clinics, and general practices. They did this through producing culturally specific advertising (e.g. posters and leaflets with relevant images and messages, but written in English; see discussion), through attending various health related and non-health related events, and through street and venue direct outreach (see Table 1). In direct outreach, the workers set up a stand outside a supermarket, for example, offering to measure exhaled carbon monoxide (CO), which naturally led to a conversation about smoking. Also, outreach workers approached Pakistani and Bangladeshi men either on the street or in workplaces. They enquired about smoking status, and talked about quitting smoking. These discussions were conducted in English or other community languages. Their aim was to refer smokers to the SSS, but literature was left with those who accepted it, even if individuals were not ready to attempt to stop smoking. Outreach workers kept a copy of referral records and checked on clinic attendance and re-referred if necessary.

**Table 1:** Description of methods used by outreach workers

Intervention phase	Methods and approaches
Phase 1 (November 2007-May 2008)	Mapping the location of existing stop smoking services and meeting with local service providers within intervention areas.
	Networking with small Asian businesses and Bangladeshi and Pakistani community organisations to promote the stop smoking services.
	Developing promotional materials for distribution e.g. posters and leaflets.
	Engaging in 'street outreach' - approaching people on main roads and side streets, signposting the stop smoking services
	Providing 'brief intervention' -counselling smokers to quit using relevant languages (English, Urdu, Mir-puri, Bengali, Sylheti), identifying suitable quit dates, distributing custom-made literature and support material (e.g. Call 2 Quit telephone number)
	Carrying out weekly follow-up and behavioural support for smokers referred on to services by telephone and SMS text messaging
	Organising promotional events at health centres and baby clinics to promote smoking cessation and highlight dangers of passive smoking to female relatives of smokers
	Accompanying health professionals (e.g. Healthy Heart workers) at events and fairs to promote stop smoking services
Phase 2 (June 2008-October 2008)	Identifying suitable venues for smoking cessation clinics
	Organising promotional events at mosques, leisure centres and libraries, with aim of raising awareness and promoting own smoking cessation clinics
	Providing smoking cessation treatment (nicotine replacement therapy) and behavioural support using relevant languages in smoking cessation clinics
	Engaging in street outreach to signpost people to own or existing smoking cessation clinics

The management team set a target of 1,500 referrals to the services in the year of the intervention, but because the actual number of referrals fell far short of this and because many of those referred did not attend for treatment the approach changed. The second phase ran for six months from June 2008 to November 2008 and concentrated on outreach workers combining more limited outreach with providing treatment for smokers directly, rather than always referring to NHS services. The outreach centred on encouraging use of a clinic that the outreach workers provided in non-NHS venues, such as barbers' shops (places of meeting for Pakistani and Bangladeshi men), mortgage brokers, taxi bases and bus depots. Sometimes these clinics were held in the evenings to overcome the problems people working shifts had in attending clinics. The revised targets were for outreach workers to treat a minimum of 10 smokers and achieve five 4-week quitters per month. Further details on the development of the two phases and outreach strategies undertaken are described in our longitudinal qualitative evaluation [26].

### Control group

Smokers living in control areas were offered NHS smoking cessation support as normal, which included advertising the availability of treatment through media campaigns. In these areas of the city, the NHS SSS consisted of healthcare service providers, including general practitioners (GPs), nurses, pharmacists and specialist NHS SSAs trained in smoking cessation, and working to standards set and monitored by the NHS SSS [34].

### Data analysis

The two primary outcomes assessed in this pilot RCT were rates of uptake of services and abstinence proportions at four weeks, and three and six months defined according to the Russell standard (i.e. using intention-to-treat and biochemical validation [35]). The uptake numerator was defined as the number of Pakistani and Bangladeshi men who set quit dates with the NHS during the intervention year that lived in the intervention and control areas. The denominator was the estimated number of Bangladeshi and Pakistani smokers in the areas. As the resultant number is an estimate rather than a true denominator, we used a Poisson multilevel model with the log of the estimated number as an offset. The rates of use of the services were estimated having adjusted for the rates of use in those areas in the 12 months prior to the intervention by including the log of these rates as a covariate. The geographical areas randomised were included in the model as a random effect, thereby allowing for the clustering inherent in the design [36].

The quit proportion was defined as the proportion of people achieving four weeks, three months, or six months prolonged abstinence allowing a standard two week grace period, with a denominator of all those who attended the service and set a quit date. Self-reporting at four weeks was verified by expired CO less than 10 parts per million [37]. We assumed that all non-responders and those who did not provide a validation sample at four weeks were still smoking. Our NHS partners were unable to carry out biochemical validation at three and six months as the protocol indicated. Consequently these data are based on self-reported prolonged abstinence. We used multilevel logistic regression models because a true denominator was available [38]. We had intended to adjust for the quit proportion in the intervention and control areas for the year prior to the intervention, but data on biochemical validation were missing at four weeks for part of the year from one PCT. Therefore these data were initially adjusted for the quit proportion achieved in the seven months prior to the intervention starting (April 2007 to end of October 2007). No baseline data were available for three and six months.

Our outcome measures examined uptake and cessation because we hoped that our intervention would affect uptake by referring more people and the success rate of those referred by supporting adherence to treatment. The net effect would thus be to increase the number of smokers stopping with NHS support and this was also measured as a population rate. The numerator was the number of people quitting smoking as defined above and the denominator was the number of smokers assessed in a Poisson model, as above, initially adjusted for the numbers stopping smoking in the seven months prior to the intervention.

The intervention had two distinct phases so, although not planned in the protocol, we examined uptake of services and 4-week quit rates by trial arm, in these two periods.

Technically, the models were fitted in SAS^® ^version 9.1, using PROC GLIMMIX. The Kenward-Roger method was used to correct for standard error bias [39].

Our process measures examined the use of smoking cessation treatments, adherence to treatments, attendance at clinic appointments and patient satisfaction with the service. We calculated the proportion of service users choosing each available treatment option. Adherence to treatments was modelled with random effects logistic regression, with the numerator being the number of people who adhered well to treatment in each week and the denominator being all those receiving treatment. Repeated measures on the same individuals were correlated within individuals and were accounted for in the analyses. The same modelling was used to calculate rates for attendance. Items from the patient satisfaction questionnaire were grouped according to convenience of service, quality of SSA and overall satisfaction. These data were analysed using χ^2 ^tests for categorical data [40]. Data collected from control and external control areas were also combined for comparison with the intervention areas.

### Health economic analysis

We adopted the perspective of the NHS as payer and assessed the costs of the intervention, with benefits and costs discounted at 3.5%. Because of the nature of the intervention, we calculated the estimated total costs and quality adjusted life years (QALYs) gained from the programme as a whole. This is different from the approach usually taken in economic evaluation of healthcare interventions delivered at the individual level, where costs and QALYs are calculated per patient. Costs such as the salary costs of the outreach workers were therefore included as fixed costs, as they did not change with the number of smokers recruited, while costs such as additional treatment costs were multiplied by the number of people treated. We modelled from the short-term abstinence rate the projected long-term abstinence rate using data from the evaluation of NHS SSS [6] and from studies with long-term follow up [41] to produce the number of lifetime abstainers. We assumed no health benefit from anything other than lifetime abstinence and we calculated an estimate of the QALYs gained using a previously developed model [42]. As quit rates are generally the primary driver of cost-effectiveness estimates [43], we used the 95% confidence interval of the rate ratio for abstinence as the only sensitivity analysis of cost-effectiveness.

## Results

### Participant flow

Based on the census, there were an estimated 14,000 Pakistani and Bangladeshi men living in the intervention areas and about 10,000 in the control areas, with 21,000 in the rest of BEN and HoB PCTs. The estimated numbers of smokers among them were 4,000, 3,000, and 7,000 respectively. Of these, 271, 169, and 524 Pakistani and Bangladeshi men in the intervention, control, and external control areas tried to stop with the support of the NHS cessation services in the year prior to the intervention (November 2006 to October 2007). The rates of service use were 63/1000 smokers/year, 58/1000/year, and 80/1000/year respectively. Of these smokers, in the seven months prior to the intervention, 63, 45, and 164 in the intervention, control, and external control areas achieved at least four weeks of biochemically confirmed abstinence. This was 25, 26, and 42/1000 smokers/year, and 44%, 42%, and 50% respectively of all smokers that set a quit date.

### Characteristics of intervention and control areas and NHS SSS users

The intervention areas had a larger population on average than the control areas, with a mean (SD) of 4,865 (6,042) compared with 3,327 (5,684) in the control areas. The socio-economic profile was similar with an IMD mean (SD) score of 56 (10) in the intervention areas and 50 (6) in the control areas. (A score of 50 or higher puts these areas in the top third most deprived in Birmingham, a city with relatively high levels of deprivation.)

The characteristics of smokers using the cessation service were also similar in the intervention and control areas (Table 2).

**Table 2:** Characteristics of all service users during the study period, and for the same period in the preceding year

	Year before study			Year of study		
	**Intervention**	**Control**	**External control**	**Intervention**	**Control**	**External control**

Number of users	271	169	524	341	163	498
Age in years mean (SD)	36.1 (12.7)	36.2 (12.1)	36.0 (12.8)	35.8 (12.0)	38.0 (14.1)	35.5 (12.0)
Ethnicity n (%)						
Bangladeshi	68 (25.1)	42 (24.9)	121 (23.1)	67 (19.6)	35 (21.5)	131 (26.3)
Pakistani	203 (74.9)	127 (75.1)	403 (76.9)	274 (80.4)	128 (78.5)	367 (73.7)

In our sub-sample of NHS SSS users, there were 52, 16 and 53 smokers who gave data to their SSAs who were resident in the intervention, control and external control areas respectively. In keeping with the population of Birmingham, most participants were Pakistani and the population were younger than seen in many smoking cessation trials in the NHS SSS and also slightly less dependent on tobacco (Table 3).

**Table 3:** Baseline characteristics of sub-sample of service users by trial arm

	Intervention	Control	External control	Combined control
All participants (n)	52	16	53	69
Age in years mean (SD)	35.8 (12.6)	34.8 (8.1)	34.2 (11.1)	34.3 (10.4)
Ethnicity n (%)				
Bangladeshi	8 (15.4)	9 (56.3)	17 (32.1)	26 (37.7)
Pakistani	44 (84.6)	7 (43.8)	36 (67.9)	43 (62.3)
Marital status n (%)				
Single	18 (34.6)	5 (31.3)	20 (37.7)	25 (36.2)
Separated	1 (1.9)	0 (0)	2 (3.8)	2 (2.9)
Married living with partner	28 (53.8)	11(68.8)	31 (58.5)	42 (60.9)
Unknown	5 (9.6)	0 (0)	0 (0)	0 (0)
Partner's smoking status n (%)				
Smoker	1 (1.9)	2 (12.5)	3 (5.7)	5 (7.2)
Non-smoker	33 (63.5)	13 (81.3)	42 (79.2)	55 (79.7)
No partner	8 (15.4)	1 (6.3)	6 (11.3)	7 (10.1)
Unknown	10 (19.2)	0 (0)	2 (3.8)	2 (2.9)
Employment				
In paid employment	18 (34.6)	11 (68.8)	27 (50.9)	38 (55.1)
Unemployed	24 (46.2)	3 (18.8)	21 (39.6)	24 (34.8)
Pensioner	0 (0)	0 (0)	1 (1.9)	1 (1.4)
Full time student	5 (9.6)	2 (12.5)	4 (7.5)	6 (8.7)
Unknown	5 (9.6)	0 (0)	0 (0)	0 (0)
Type of Work n (%)				
Manual	29 (55.8)	9 (56.3)	37 (69.8)	46 (66.7)
Clerical secretarial	4 (7.7)	0 (0)	3 (5.7)	3 (4.3)
Managerial professional	6 (11.5)	6 (37.5)	3 (5.7)	9 (13.0)
Not worked	5 (9.6)	1 (6.3)	6 (11.3)	7 (10.1)
Unknown	8 (15.4)	0 (0)	4 (7.5)	4 (5.8)
Highest Education n (%)				
None	14 (26.9)	6 (37.5)	15 (28.3)	21 (30.4)
GCSE or equivalent	16 (30.8)	5 (31.3)	17 (32.1)	22 (31.9)
A-level or equivalent	8 (15.4)	2 (12.5)	10 (18.9)	12 (17.4)
Degree or equivalent	5 (9.6)	2 (12.5)	6 (11.3)	8 (11.6)
Other	3 (5.8)	0 (0)	5 (9.4)	5 (7.2)
Unknown	6 (11.5)	1 (6.3)	0 (0)	1 (1.4)
FTND* mean (SD)	4.4 (2.7)	4.4 (2.1)	4.6 (2.4)	4.6 (2.3)
Age of starting smoking in years mean (SD)	17.6 (6.5)	18.1 (4.3)	17.6 (5.2)	17.7 (5.0)
Cigarettes per day mean (SD)	15 (10)	16 (5)	17 (7)	17 (7)
Number past quit attempts mean (SD)	1 (1)	1 (1)	1 (1)	1 (1)
Maximum length of previous quit attempt in days, median (range)	21 (1-336)	14 (1-168)	21 (1-672)	21 (1-672)

*Fagerstrom Test for Nicotine Dependence, scored from 0-10.

### Implementation of the intervention

In phase one of the intervention, outreach workers discussed smoking cessation with 1,916 people (smokers and non-smokers), of whom 229 (12%) smokers accepted referral to the cessation service, and 58 (3%) attempted cessation. In phase two, outreach workers approached 1,733 people, where 164 (9%) smokers were referred to the cessation service, of whom at least 38 (2%) were treated by outreach workers.

### Effect of intervention on rate of use of NHS SSS

**Figure 1:**
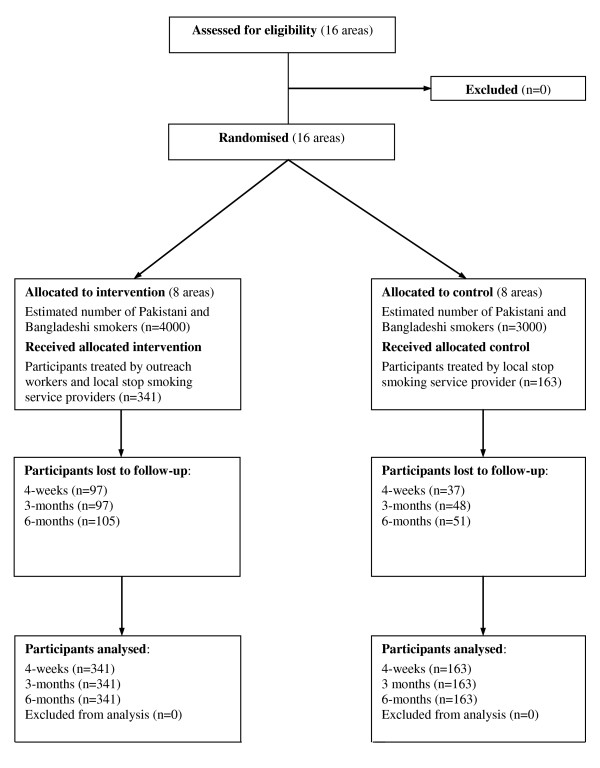
**Flow diagram of clusters and participants in the trial**.

During the intervention year, the absolute use of the NHS SSS by residents of control areas decreased by six to 163 and the rate declined slightly to 56/1000 smokers/year (Figure 1). We might therefore have expected little change among the intervention areas, but there was an increase of 70 to 341 with the rate climbing from 63 to 80/1000 smokers/year. The rate ratio (RR) and 95% confidence intervals (CI) for service use versus the internal control was 1.32 (95%CI 1.03, 1.69). There was also a small decline in the external control area of 26 to 498 users, a rate of 76/1000 smokers/year. The RR for intervention relative to external control was 1.28 (95%CI 1.02, 1.60).

### Quit proportions and quit rates

There was almost no change in the proportion of smokers achieving four weeks of abstinence confirmed by CO in the control group from the period seven months prior to the intervention to the period of the intervention: the percentages were 43% prior to and during the intervention period. In the intervention areas, the percentage achieving abstinence fell slightly in the intervention period, from 44% to 39%. The intention was to adjust for secular trends, but across areas there was a weak negative association between the percentages in successive years so no adjustment was made. The odds ratio (OR) was 0.86 (95%CI 0.52, 1.42) relative to control areas. In the external control group, the proportion that was successful declined from 50% to 41% and the OR for intervention relative to the external control group was 0.92 (95%CI 0.61, 1.38). At three months, 48 (14%) of the intervention area quitters reported prolonged abstinence and 38 (11%) did so at six months. The corresponding numbers for the control areas were 25 (15%) and 14 (9%). The ORs of self-reported prolonged abstinence at three and six months for the intervention versus the control were 1.04 (95%CI 0.40, 2.66) and 1.61 (95%CI 0.50, 5.17). No prior data were available. Versus the external controls, the ORs were 1.27 (95%CI 0.43, 3.68) for the intervention group and 1.49 (95%CI 0.41, 5.43) for the control group.

The effects on population four-week abstinence rates combine the uptake rates and outcome of quit attempts. The control rate decreased slightly from 26 to 24/1000 smokers/year and the intervention rate increased from 25 to 31/1000 smokers/year. The rate ratio for intervention versus control, unadjusted for secular trends was 1.30 (95%CI 0.82, 2.06). In the external controls, the rate of abstinence declined from 42 to 31/1000 smokers/year and the rate ratio of the intervention group relative to this was 1.00 (95%CI 0.69, 1.45).

### Adherence to treatments, attendance rates and patient satisfaction

In our sub-sample of NHS SSS users, nicotine patches were used more often than any other form of nicotine replacement therapy (NRT) in the intervention (61.5%), control (56.3%) and external control (73.8%) areas. No differences were found between groups in the rate at which people adhered well to treatments at each clinic appointment (Table 4). The proportion of people attending weekly clinic appointments was lower in intervention areas compared to control and external control areas, with less than a third of participants returning for their subsequent sessions (Table 5).

**Table 4:** Adherence to treatments in sub-sample of service users by trial arm

Sessions of behavioural support	n (%) good adherence				Intervention versus control		
	**Intervention**	**Control**	**External control**	**Combined control**	**Intervention vs control** **RR (95%CI)**	**Intervention vs external control** **RR (95%CI)**	**Intervention vs combined control** **RR (95%CI)**

Session 1	51 (98.1)	16 (100)	52 (98.1)	68 (98.6)	0.98 (0.94-1.02)	1.00 (0.95-1.05)	1.00 (0.95-1.04)
Session 2	11 (73.3)	3 (60.0)	14 (82.4)	17 (77.3)	1.22 (0.56-2.66)	0.89 (0.61-1.30)	0.95 (0.65-1.39)
Session 3	10 (76.9)	3 (60.0)	7 (77.8)	10 (71.4)	1.28 (0.59-2.78)	0.99 (0.63-1.56)	1.08 (0.69-1.68)
Session 4	6 (75.0)	4 (80.0)	6 (100.0)	10 (71.4)	0.94 (0.52-1.70)	1.00 (0.57-1.76)	0.97 (0.59-1.61)
Session 5	5 (83.3)	0 (0)	5 (55.6)	5 (55.6)	-	1.00 (0.60-1.66)	1.50 (0.76-2.98)

**Table 5:** Attendance at weekly clinics in sub-sample of service users by trial arm

Sessions of behavioural support	n (%) attendance				Intervention versus control		
	**Intervention**	**Control**	**External control**	**Combined control**	**Intervention vs control** **RR (95%CI)**	**Intervention vs external control RR (95%CI)**	**Intervention vs combined control** **RR (95%CI)**

Session 1	52 (100)	16 (100)	53 (100)	69 (100)	1	1	1
Session 2	15 (28.8)	5 (31.3)	17 (32.1)	22 (31.9)	0.92 (0.40-2.14)	0.90 (0.50-1.61)	0.90 (0.52-1.57)
Session 3	13 (25.0)	5 (31.3)	9 (17.0)	14 (20.3)	0.80 (0.34-1.90)	1.47 (0.69-3.14)	1.23 (0.63-2.39)
Session 4	8 (15.4)	5 (31.3)	6 (11.3)	13 (18.8)	0.49 (0.19-1.29)	1.02 (0.41-2.51)	0.82 (0.37-1.82)
Session 5	6 (11.5)	3 (18.8)	8 (15.1)	9 (13.0)	0.62 (0.17-2.19)	1.02 (0.35-2.96)	0.88 (0.34-2.33)

Most consultations were carried out in English in the intervention (61.5%), control (62.5%) and external control (67.9%) areas, although outreach workers used a combination of English with another language (25.8%) more frequently than SSAs (15.3%). These data are available from the corresponding author.

At three-month follow-up, patient satisfaction data were collected from 38 (30.6%) service users in our sub-sample. No differences were found between the intervention and combined control in the convenience of the service offered, the quality of SSA or overall satisfaction (Table 6).

**Table 6:** Patient satisfaction in sub-sample of service users by trial arm

	Intervention	Combined control	Intervention versus combined control	
	**median (range)**		**χ2**	**p Value**

Variable				
Convenience of service†	13 (9-15)	12 (6-15)	1.39*	0.24
Quality of stop smoking advisor‡	28 (23-35)	31 (19-35)	0.49*	0.49
Overall satisfaction§	8 (6-9)	8 (5-9)	0.64*	0.42

†Three items related to convenience of service. Response categories ranged from 1 (very inconvenient/very dissatisfied) to 5 (very convenient/very satisfied).

‡Five items related to quality of stop smoking advisor. Response categories ranged from 1 (very dissatisfied) to 5 (very satisfied).

§Two items related to overall satisfaction. Response categories ranged from 1 (very dissatisfied) to 5 (very satisfied) and 1 (no would not recommend service) to 4 (yes would recommend service wholeheartedly).

*χ2 Test for trend.

### Ancillary analyses

As the intervention developed in two phases, we examined for evidence that the effect of the intervention relative to the control and external control varied by phase. There was no strong evidence for this (Table 7).

**Table 7:** Comparison of rates of setting quit dates and achieving 4-week quit rates between the trial arms during the two phases of the trial

	Setting quit date		Russell standard abstinence at 4 weeks	
	
	RR (95%CI)	P-value	RR (95%CI)	P-value
As a rate of all smokers				
Randomised Comparison				
Overall	1.32 (1.03, 1.69)	0.03	1.30 (0.82, 2.06)	0.24
1st Phase	1.24 (0.88, 1.75)	0.21	1.36 (0.78, 2.36)	0.25
2nd Phase	1.57 (1.03, 2.41)	0.04	1.25 (0.71, 2.22)	0.42
				
				
Versus External Controls				
Overall	1.28 (1.02, 1.60)	0.03	1.00 (0.69, 1.45)	0.99
1st Phase	1.06 (0.81, 1.39)	0.66	0.94 (0.60, 1.47)	0.78
2nd Phase	1.36 (0.97, 1.91)	0.07	1.09 (0.67, 1.78)	0.70
Interaction test		0.42		0.70
				
As proportion of those setting quit date			OR (95%CI)	P-value
			
Randomised comparison				
Overall			0.86 (0.52, 1.42)	0.53
1st Phase			1.06 (0.58, 1.94)	0.85
2nd Phase			0.60 (0.30, 1.20)	0.14
				
Versus External Controls				
Overall			0.92 (0.61, 1.38)	0.66
1st Phase			0.96 (0.59, 1.56)	0.85
2nd Phase			0.84 (0.48, 1.47)	0.51
Interaction test				0.34

### Cost-effectiveness

The point estimate of the rate ratio for service use implies that the effect of the intervention was to increase the number of smokers trying to quit by 83, after adjustment for secular trends. Applying the intervention four-week abstinence rates to this number yielded an additional 32 achieving four-week confirmed abstinence, which we estimated resulted in an additional 5.6 lifetime abstainers applying relapse rates to one year [6] and beyond [41]. Using a previous model [42], we estimated this would yield an additional 14.6 QALYs. The total cost of the intervention to achieve this was £124,000; an estimated cost per QALY gained of £8,500. Applying the upper limit of the 95% confidence interval gave an estimated cost/QALY gained of £2,000. Applying the lower limit for the rate ratio for increased use resulted in an estimated cost/QALY gained of over £100,000.

## Discussion

This pilot trial has demonstrated that it was possible to randomise geographical areas and deliver the NHS SSS outreach-based model of care to Pakistani and Bangladeshi smokers. The effect of the intervention was to increase the rate of uptake by approximately 30%, although this was imprecisely estimated. The number of smokers achieving abstinence as a proportion of all those trying to quit in the intervention areas was lower than in the control areas, although the confidence intervals of the odds ratio encompassed unity. This may have been a result of our recruiting in the intervention areas smokers with lower motivation to quit using the service. Retention in the behavioural support programme was somewhat lower for outreach workers than for typical SSS providers, which further suggests that relatively unmotivated smokers were drawn into the service. Treatment preference, adherence to treatments and satisfaction with the cessation service was the same across all areas.

Overall, there was evidence of a clinically relevant 30% change in the number of abstinent smokers, but, as might be expected from a pilot trial, this was not statistically significant. The most likely estimate of the cost-effectiveness of this intervention would make this intervention highly cost-effective by typical NHS standards [44], but the imprecision of the estimate precludes firm conclusions. These data suggest that a full trial may well be justified, timely and feasible.

The need for a full trial should, however, be considered in relation to the current context. When we planned the intervention, we estimated that South Asian smokers were using the NHS services at only half the rate of the rest of the population. Since then, the NHS SSS have improved their data collection and now it is possible to provide ethnic-specific rates. In 2007/8 in England, the rate of use of the NHS SSS was 68/1000 smokers in White British and 45/1000 in Pakistanis and 56/1000 Bangladeshi smokers. In 2008/9, the rates were 67/1000 in White British, 54/1000 in Pakistanis, and 82/1000 in Bangladeshis. In 2006/7, in our intervention and control areas, the rates of use were 63/1000 and 58/1000 and after the intervention in 2007/8 were 80/1000 and 56/1000, suggesting that service use by Pakistani and Bangladeshi smokers in Birmingham might have been somewhat higher than the national average before the intervention. There was very little variation in the English data on quit rates by ethnic group as a proportion of all those attending. Another way to consider this is that the RR for the increase in service use in England as a whole for 2008/9 versus 2007/8 were 1.19 (95%CI 1.15, 1.24) for Pakistanis and 1.48 (95%CI 1.41, 1.55) for Bangladeshis. These data indicate that, largely without outreach workers, NHS services managed to increase their reach into the Pakistani and Bangladeshi communities and rates of use by these groups are now similar to or higher than the White British population nationally.

Our results are similar to another trial, which sought to raise the use of the NHS SSS [15]. Our results also suggested the intervention seemed to raise throughput, though somewhat at the expense of lowered quit proportion, with no statistically significant effect overall.

Although our intervention targeted Pakistanis and Bangladeshis, interventions with each group were separate, usually in different venues, communicating with people in their preferred languages. Our outreach workers, for example, produced a range of advertising materials to promote themselves and the cessation services. They carried out focus groups with members of the public, community networks and pharmacists to determine the suitability and relevance of all materials. Most people they spoke to thought that it would not be effective to have promotional materials written in community languages. They held the view that while many Pakistanis and Bangladeshis speak in languages particular to their ethnic group, many of these same people cannot read or write in their own languages. All advertising materials were therefore written in English, but they contained culturally relevant images and messages. The outreach workers did not suggest that the intervention was effective only with one group and not the other in their exit interviews [26]. We combined the results for the two ethnic groups as we saw no reason not to do so. Our qualitative work [26] suggested that Pakistani and Bangladeshi smokers shared many common issues with respect to their smoking and stopping smoking, as found in other qualitative studies [13,45]. Any future trial might therefore reasonably address both groups in the way that we did.

The strength of this study is that it is one of the few randomised controlled trials of community interventions to increase smoking cessation rates in minority and socio-economically deprived populations. As such it provides unbiased estimates of the effects of these interventions. However, it was a preliminary study and the small sample size in the study precludes definitive conclusions on the effectiveness and cost-effectiveness of the model. Also, the study took place in one city with areas nearby to one another randomised to intervention and control status. Participants were defined by their postcode of residence and a number of people who lived in control areas were beneficiaries of the intervention and this effect would reduce the apparent benefit of the intervention. A further issue is that the year of observation prior to the intervention included the change to prohibit smoking in all indoor public places and this had a small effect on increasing use of NHS SSS [11], though this would increase the rate of use of the service in both intervention and control areas and not bias the rate ratio. This effect might have led to the small decline in use from before the intervention to during it seen in the control area.

The aim of this study was to prepare for a definitive phase III trial and the question arises as to whether we know enough to take this forward. In favour of this would be the effect estimates, which show an approximate 30% increase in rate of use of the NHS SSS and quit rates in socio-economically deprived populations at high risk of cardiovascular disease. In favour too would be the potentially low cost/QALY. However, NHS services may view this balance differently. Four workers working for one year managed to encourage another 83 people to stop smoking with NHS support, or approximately one person every 2-3 weeks/worker. The workers found the process of raising awareness and referring smokers to the service dispiriting because the large majority of people referred failed to attend. However, they were more encouraged by running their own clinics [26], but the results suggest little difference in the effectiveness of these two approaches.

There is a similar and long-running outreach programme in Tower Hamlets in London, the borough with the highest proportion of Bangladeshi residents in England. This service treated 463 male smokers in 2008/9 out of a total of 1,104 (42%) in Tower Hamlets [46]. Given these data and the data on the increasing use of services by Pakistani and Bangladeshi smokers nationally, it is clear that outreach might have a role to play, but the mainstay of reaching these smokers is attracting them to general NHS services and the NHS has been successful at doing so over the past year.

## Conclusions

This pilot cluster randomised controlled trial produced evidence that outreach workers can encourage Pakistani and Bangladeshi smokers to use NHS support for cessation. The increase in use and increase in population quit rates obtained suggests that this kind of intervention is worth evaluating in a definitive trial. Our experiences from running this pilot trial, the data now available for sample size calculations and the insights gained from the accompanying qualitative work should prove valuable in planning for our formal randomised controlled trial.

## Ethical approval

This study has been approved by South Staffordshire Local Research Ethics Committee.

## Abbreviations

BEN: Birmingham East and North Primary Care Trust; HoBt: Heart of Birmingham Teaching Primary Care Trust; NHS: National Health Service; NRT: Nicotine Replacement Therapy; PCTs: Primary Care Trusts; QALY: Quality Adjusted Life Years; SOA: Super Output Area; SSA: Stop Smoking Advisor; SSS: Stop Smoking Services.

## Competing interests

PA has done consultancy work for Pfizer, McNeil, and Xenva (now Celtic) Biotechnology with regard to smoking cessation. All other authors declare that they have no competing interests.

## Authors' contributions

AS and RSB conceived this study and together led the bid to secure funding for this work. PU, RAB, PA, and AS managed the study. RSB, MW, AA, RJP, RB, PB, PG, QZ and MF all contributed to designing the study and overseeing its implementation. RJP carried out the statistical analyses. RAB and PA drafted the paper and all authors commented on draft versions of this manuscript and approved the final version prior to submission. The authors had access to the data and accept full responsibility for the conduct of the study. All authors are the study guarantors.
